# 2-(4-Chloro­benzamido)­acetic acid

**DOI:** 10.1107/S1600536811009536

**Published:** 2011-03-19

**Authors:** Islam Ullah Khan, Muneeb Hayat Khan, Muhammad Nadeem Arshad, Mehmet Akkurt

**Affiliations:** a Materials Chemistry Laboratory, Department of Chemistry, GC University, Lahore 54000, Pakistan; b Department of Physics, Faculty of Sciences, Erciyes University, 38039 Kayseri, Turkey

## Abstract

In the crystal structure of the title mol­ecule, C_9_H_8_ClNO_3_, adjacent mol­ecules are arranged into centrosymmetric dimers through pairs of inter­molecular O—H⋯O inter­actions. Inter­molecular N—H⋯O hydrogen bonds link the dimers into a layer parallel to the *bc* plane. In the layer, mol­ecules are packed in a face-to-face π-stacked arrangment, showing π–π stacking inter­actions between the benzene rings with a centroid–centroid distance of 3.6884 (8) Å.

## Related literature

For crystallographic studies of benzamide derivatives, see: Donnelly *et al.* (2008 ▶); Mugnoli *et al.* (1991 ▶); Stensland *et al.* (1995 ▶). For standard bond lengths, see: Allen *et al.* (1987 ▶).
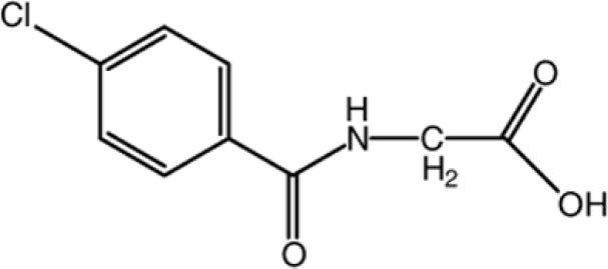


## Experimental

### 

#### Crystal data

C_9_H_8_ClNO_3_
*M* *_r_* = 213.61
Monoclinic, 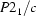

*a* = 10.5035 (2) Å
*b* = 13.2105 (4) Å
*c* = 7.1226 (2) Å
β = 102.203 (1)°
*V* = 965.98 (4) Å^3^
*Z* = 4
Mo *K*α radiation
μ = 0.37 mm^−1^
*T* = 296 K
0.36 × 0.21 × 0.13 mm

#### Data collection

Bruker APEXII CCD diffractometer
9027 measured reflections
2365 independent reflections
1627 reflections with *I* > 2σ(*I*)
*R*_int_ = 0.028

#### Refinement

*R*[*F* ^2^ > 2σ(*F* ^2^)] = 0.035
*wR*(*F* ^2^) = 0.098
*S* = 1.02
2365 reflections
133 parameters
2 restraints
H atoms treated by a mixture of independent and constrained refinement
Δρ_max_ = 0.21 e Å^−3^
Δρ_min_ = −0.25 e Å^−3^

### 

Data collection: *APEX2* (Bruker, 2007 ▶); cell refinement: *SAINT* (Bruker, 2007 ▶); data reduction: *SAINT*; program(s) used to solve structure: *SHELXS97* (Sheldrick, 2008 ▶); program(s) used to refine structure: *SHELXL97* (Sheldrick, 2008 ▶); molecular graphics: *ORTEP-3 for Windows* (Farrugia, 1997 ▶); software used to prepare material for publication: *WinGX* (Farrugia, 1999 ▶) and *PLATON* (Spek, 2009 ▶).

## Supplementary Material

Crystal structure: contains datablocks global, I. DOI: 10.1107/S1600536811009536/is2688sup1.cif

Structure factors: contains datablocks I. DOI: 10.1107/S1600536811009536/is2688Isup2.hkl

Additional supplementary materials:  crystallographic information; 3D view; checkCIF report

## Figures and Tables

**Table 1:** Hydrogen-bond geometry (Å, °)

*D*—H⋯*A*	*D*—H	H⋯*A*	*D*⋯*A*	*D*—H⋯*A*
N1—H1*N*⋯O2^i^	0.83 (2)	2.06 (2)	2.8491 (19)	160 (2)
O3—H1*O*⋯O1^ii^	0.83 (1)	1.85 (2)	2.6613 (16)	165 (2)

Symmetry codes: (i) 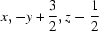; (ii) 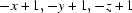.

**Fig. 1.**
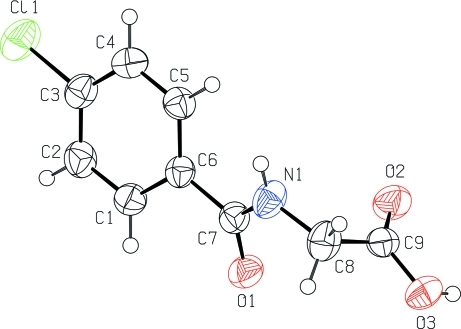
The title molecule with atom numbering scheme. Displacement ellipsoids for non-H atoms are drawn at the 50% probability level.

**Fig. 2.**
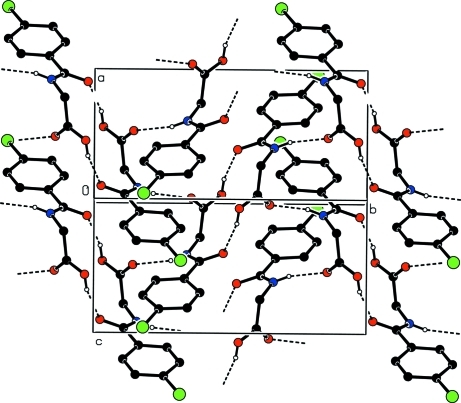
View of the centrosymmetric dimers forming through a pair of O—H···O interactions which are connected to each other through intermolecular N—H···O interactions. Hydrogen atoms not involved in hydrogen bonding have been omitted for clarity.

## supplementary crystallographic information

### Comment

Benzamide is originally a derivative of benzoic acid. Some benzamide derivatives are in use as Analgesics (Ethenzamide, Salicylamide), Antiemetics/Prokinetics (Alizapride, Bromopride, Cinitapride, Cisapride, Clebopride) and Antipsychotics (Amisulpride, Nemonapride, Remoxipride, Sulpiride, Sultopride). Other benzamides are being prepared and there crystallographic studies are done (Donnelly *et al.*, 2008; Stensland *et al.*, 1995; Mugnoli *et al.*, 1991). The given benzamide derivative was prepared using the simple route using water as solvent.

In the title compound (I), (Fig. 1), the bond lengths and bond angles are in agreement with those reported in the literature (Allen *et al.*, 1987). The C1—C6—C7—O1, C1—C6—C7—N1, O1—C7—N1—C8, N1—C8—C9—O2 and N1—C8—C9—O3 torsion angles are 20.2 (2), -159.08 (14), -3.2 (2), 17.7 (2) and -163.65 (14)°, respectively.

In the crystal structure, the molecules adopt a face-to-face π-stacked packing arrangement showing π–π stacking interactions involving the benzene rings [*Cg*1···*Cg*1*^i^* = 3.6884 (8) Å; symmetry code: (i) *x*, 3/2 - *y*, -1/2 + *z*; *Cg*1 is a centroid of the benzene ring (C1–C6)].

### Experimental

The calculated amount of glycine (0.5 g, 6.494 mmol) was carefully weighed and transferred to R.B.F (50 ml) containing 10 ml of distilled water. The pH of the water was maintained at 8 with 10% Sod. Carbonate solution which results in the complete dissolution of glycine. Then 4-chlorobenzoyl chloride (0.83 ml, 6.494 mmol) was added and pH was maintained at 8. After 3.5 h the TLC showed the completion of reaction giving a single spot of the product. The reaction mixture was then acidified with 3 N HCl up to pH 3 which resulted in the insoluble precipitate formation. Precipitates were filtered, washed, dried and then crystallized in methanol.

### Refinement

In the last cycles of the refinement, 2 reflections (1 0 0) and (0 2 0) were eliminated due to being poorly measured in the vicinity of the beam stop. H atoms bounded to C atoms were positioned geometrically with C—H = 0.93 and 0.97 Å, and allowed to ride on their parent atoms, with *U*_iso_(H) = 1.2*U*_eq_(C). The hydroxyl and amine H atoms were located in a difference Fourier map, and refined with the distance restraints N—H = 0.86 (2) Å and O—H = 0.82 (2) Å. Their isotropic displacement parameters were set to be 1.2*U*_eq_(N) for amine and 1.5*U*_eq_(O) for hydroxyl.

### Crystal data

**Table:**

C_9_H_8_ClNO_3_	*F*(000) = 440
*M**_r_* = 213.61	*D*_x_ = 1.469 Mg m^−^^3^
Monoclinic, *P*2_1_/*c*	Mo *K*α radiation, λ = 0.71073 Å
Hall symbol: -P 2ybc	Cell parameters from 3071 reflections
*a* = 10.5035 (2) Å	θ = 2.5–26.5°
*b* = 13.2105 (4) Å	µ = 0.37 mm^−^^1^
*c* = 7.1226 (2) Å	*T* = 296 K
β = 102.203 (1)°	Needle, colourless
*V* = 965.98 (4) Å^3^	0.36 × 0.21 × 0.13 mm
*Z* = 4	

### Data collection

**Table:**

Bruker APEXII CCD diffractometer	1627 reflections with *I* > 2σ(*I*)
Radiation source: sealed tube	*R*_int_ = 0.028
graphite	θ_max_ = 28.3°, θ_min_ = 3.3°
φ and ω scans	*h* = −13→13
9027 measured reflections	*k* = −17→17
2365 independent reflections	*l* = −9→9

### Refinement

**Table:**

Refinement on *F*^2^	Primary atom site location: structure-invariant direct methods
Least-squares matrix: full	Secondary atom site location: difference Fourier map
*R*[*F*^2^ > 2σ(*F*^2^)] = 0.035	Hydrogen site location: inferred from neighbouring sites
*wR*(*F*^2^) = 0.098	H atoms treated by a mixture of independent and constrained refinement
*S* = 1.02	*w* = 1/[σ^2^(*F*_o_^2^) + (0.0427*P*)^2^ + 0.1658*P*] where *P* = (*F*_o_^2^ + 2*F*_c_^2^)/3
2365 reflections	(Δ/σ)_max_ = 0.001
133 parameters	Δρ_max_ = 0.21 e Å^−^^3^
2 restraints	Δρ_min_ = −0.24 e Å^−^^3^

### Special details

**Table:**

Geometry. Bond distances, angles *etc*. have been calculated using the rounded fractional coordinates. All su's are estimated from the variances of the (full) variance-covariance matrix. The cell e.s.d.'s are taken into account in the estimation of distances, angles and torsion angles
Refinement. Refinement on *F*^2^ for ALL reflections except those flagged by the user for potential systematic errors. Weighted *R*-factors *wR* and all goodnesses of fit *S* are based on *F*^2^, conventional *R*-factors *R* are based on *F*, with *F* set to zero for negative *F*^2^. The observed criterion of *F*^2^ > σ(*F*^2^) is used only for calculating -*R*-factor-obs *etc*. and is not relevant to the choice of reflections for refinement. *R*-factors based on *F*^2^ are statistically about twice as large as those based on *F*, and *R*-factors based on ALL data will be even larger.

### Fractional atomic coordinates and isotropic or equivalent isotropic displacement parameters (Å^2^)

**Table:**

	*x*	*y*	*z*	*U*_iso_*/*U*_eq_	
Cl1	1.15861 (4)	0.81838 (4)	0.19567 (7)	0.0678 (2)	
O1	0.65691 (11)	0.52525 (8)	0.26411 (16)	0.0518 (4)	
O2	0.46720 (11)	0.64368 (9)	0.49155 (17)	0.0576 (4)	
O3	0.31367 (11)	0.53263 (9)	0.37136 (17)	0.0519 (4)	
N1	0.53959 (13)	0.65903 (11)	0.1375 (2)	0.0515 (5)	
C1	0.88594 (15)	0.60968 (12)	0.1921 (2)	0.0457 (5)	
C2	1.00353 (16)	0.65563 (13)	0.1908 (2)	0.0499 (6)	
C3	1.00957 (15)	0.75964 (13)	0.1880 (2)	0.0453 (5)	
C4	0.90087 (16)	0.81827 (12)	0.1838 (2)	0.0465 (5)	
C5	0.78344 (15)	0.77170 (11)	0.1832 (2)	0.0423 (5)	
C6	0.77497 (14)	0.66710 (11)	0.1887 (2)	0.0379 (4)	
C7	0.65311 (14)	0.61215 (11)	0.2002 (2)	0.0407 (5)	
C8	0.41924 (16)	0.61044 (15)	0.1527 (2)	0.0539 (6)	
C9	0.40478 (14)	0.59869 (11)	0.3568 (2)	0.0411 (5)	
H1	0.88100	0.53940	0.19530	0.0550*	
H1N	0.538 (2)	0.7167 (13)	0.092 (3)	0.0810*	
H1O	0.315 (2)	0.5241 (19)	0.487 (2)	0.1020*	
H2	1.07760	0.61690	0.19170	0.0600*	
H4	0.90650	0.88850	0.18130	0.0560*	
H5	0.70930	0.81080	0.17910	0.0510*	
H8A	0.41540	0.54410	0.09330	0.0650*	
H8B	0.34680	0.65000	0.08250	0.0650*	

### Atomic displacement parameters (Å^2^)

**Table:**

	*U*^11^	*U*^22^	*U*^33^	*U*^12^	*U*^13^	*U*^23^
Cl1	0.0478 (3)	0.0785 (4)	0.0752 (3)	−0.0209 (2)	0.0086 (2)	−0.0017 (2)
O1	0.0541 (7)	0.0422 (7)	0.0616 (7)	0.0003 (5)	0.0180 (5)	0.0100 (5)
O2	0.0571 (8)	0.0578 (7)	0.0566 (7)	−0.0178 (6)	0.0089 (6)	−0.0106 (6)
O3	0.0439 (6)	0.0499 (7)	0.0600 (7)	−0.0122 (5)	0.0067 (5)	0.0017 (6)
N1	0.0389 (8)	0.0556 (9)	0.0604 (9)	0.0027 (6)	0.0113 (6)	0.0169 (7)
C1	0.0449 (9)	0.0391 (8)	0.0547 (9)	0.0039 (7)	0.0141 (7)	0.0045 (7)
C2	0.0392 (9)	0.0546 (10)	0.0566 (10)	0.0065 (7)	0.0119 (7)	0.0016 (7)
C3	0.0414 (9)	0.0532 (10)	0.0401 (8)	−0.0082 (7)	0.0060 (6)	−0.0003 (7)
C4	0.0524 (10)	0.0391 (8)	0.0461 (9)	−0.0040 (7)	0.0061 (7)	0.0011 (7)
C5	0.0431 (9)	0.0409 (8)	0.0425 (8)	0.0065 (6)	0.0081 (6)	0.0036 (6)
C6	0.0395 (8)	0.0407 (8)	0.0338 (7)	0.0019 (6)	0.0084 (6)	0.0048 (6)
C7	0.0435 (9)	0.0406 (9)	0.0394 (8)	0.0018 (6)	0.0117 (6)	0.0035 (6)
C8	0.0374 (9)	0.0698 (12)	0.0530 (10)	−0.0044 (8)	0.0065 (7)	−0.0001 (8)
C9	0.0300 (8)	0.0351 (8)	0.0568 (9)	0.0026 (6)	0.0062 (7)	−0.0004 (7)

### Geometric parameters (Å, °)

**Table:**

Cl1—C3	1.7377 (17)	C3—C4	1.375 (2)
O1—C7	1.2325 (18)	C4—C5	1.378 (2)
O2—C9	1.1993 (19)	C5—C6	1.386 (2)
O3—C9	1.3153 (19)	C6—C7	1.489 (2)
O3—H1O	0.829 (14)	C8—C9	1.501 (2)
N1—C8	1.442 (2)	C1—H1	0.9300
N1—C7	1.334 (2)	C2—H2	0.9300
N1—H1N	0.827 (18)	C4—H4	0.9300
C1—C2	1.378 (2)	C5—H5	0.9300
C1—C6	1.387 (2)	C8—H8A	0.9700
C2—C3	1.376 (2)	C8—H8B	0.9700
			
C9—O3—H1O	108.0 (16)	N1—C8—C9	112.86 (13)
C7—N1—C8	120.26 (14)	O2—C9—O3	123.33 (14)
C7—N1—H1N	120.1 (15)	O2—C9—C8	125.00 (14)
C8—N1—H1N	119.6 (15)	O3—C9—C8	111.66 (13)
C2—C1—C6	120.68 (15)	C2—C1—H1	120.00
C1—C2—C3	118.96 (15)	C6—C1—H1	120.00
C2—C3—C4	121.49 (15)	C1—C2—H2	120.00
Cl1—C3—C2	119.31 (13)	C3—C2—H2	121.00
Cl1—C3—C4	119.18 (13)	C3—C4—H4	120.00
C3—C4—C5	119.17 (15)	C5—C4—H4	120.00
C4—C5—C6	120.53 (14)	C4—C5—H5	120.00
C1—C6—C5	119.16 (14)	C6—C5—H5	120.00
C1—C6—C7	117.49 (13)	N1—C8—H8A	109.00
C5—C6—C7	123.29 (14)	N1—C8—H8B	109.00
N1—C7—C6	118.25 (13)	C9—C8—H8A	109.00
O1—C7—N1	120.86 (14)	C9—C8—H8B	109.00
O1—C7—C6	120.89 (13)	H8A—C8—H8B	108.00
			
C8—N1—C7—C6	−177.54 (13)	C3—C4—C5—C6	0.6 (2)
C7—N1—C8—C9	67.7 (2)	C4—C5—C6—C7	176.22 (13)
C8—N1—C7—O1	3.2 (2)	C4—C5—C6—C1	−0.9 (2)
C2—C1—C6—C7	−177.03 (13)	C1—C6—C7—O1	20.2 (2)
C2—C1—C6—C5	0.3 (2)	C1—C6—C7—N1	−159.08 (14)
C6—C1—C2—C3	0.6 (2)	C5—C6—C7—O1	−156.98 (14)
C1—C2—C3—Cl1	177.54 (11)	C5—C6—C7—N1	23.8 (2)
C1—C2—C3—C4	−0.9 (2)	N1—C8—C9—O2	16.7 (2)
Cl1—C3—C4—C5	−178.18 (11)	N1—C8—C9—O3	−163.65 (14)
C2—C3—C4—C5	0.3 (2)		

### Hydrogen-bond geometry (Å, °)

**Table:**

*D*—H···*A*	*D*—H	H···*A*	*D*···*A*	*D*—H···*A*
N1—H1N···O2^i^	0.83 (2)	2.06 (2)	2.8491 (19)	160 (2)
O3—H1O···O1^ii^	0.83 (1)	1.85 (2)	2.6613 (16)	165 (2)

Symmetry codes: (i) *x*, −*y*+3/2, *z*−1/2; (ii) −*x*+1, −*y*+1, −*z*+1.
